# Berberine Ameliorates Spatial Learning Memory Impairment and Modulates Cholinergic Anti-Inflammatory Pathway in Diabetic Rats

**DOI:** 10.3389/fphar.2019.01003

**Published:** 2019-09-06

**Authors:** Kaifu Wang, Qingjie Chen, Ninghua Wu, Yong Li, Ruyi Zhang, Jiawen Wang, Di Gong, Xin Zou, Chao Liu, Juan Chen

**Affiliations:** ^1^Institute of Integrated Traditional Chinese and Western Medicine, Tongji Hospital, Tongji Medical College, Huazhong University of Science and Technology, Wuhan, China; ^2^Hubei Key Laboratory of Genetics and Molecular Mechanisms of Cardiological Disorders, Hubei University of Science and Technology, Xianning, China; ^3^Basic Medical College, Hubei University of Science and Technology, Xianning, China; ^4^Department of Biochemistry and Molecular Biology, School of Basic Medicine and the Collaborative Innovation Center for Brain Science, Tongji Medical College, Huazhong University of Science and Technology, Wuhan, China; ^5^Key Laboratory of Neurological Disease of National Education Ministry, Tongji Medical College, Huazhong University of Science and Technology, Wuhan, China

**Keywords:** berberine, α7nAChRs, diabetes mellitus, spatial learning and memory, inflammation, Aβ

## Abstract

**Background:** Cognitive impairment caused by diabetes has been recognized. Berberine is well known for its resistance to peripheral lesions, but it is rarely used for the treatment of spatial learning and memory caused by diabetes. This study explored the mechanism of berberine to alleviate cognitive impairment *via* the cholinergic anti-inflammatory and insulin signaling pathways.

**Methods:** Morris water maze was used to appraise spatial learning and memory. Positron-emission tomography (PET) imaging was adopted to detect the transport of glucose, and blood/cerebrospinal fluid (CSF) glucose was checked using commercial blood glucose meter. Insulin level was measured by ELISA kit and β-Amyloid (Aβ) formation was observed by Congo red staining. Western-blot was performed to appraise protein expression.

**Results:** We found that berberine rectified some aberrant changes in signal molecules concerning inflammation, and cholinergic and insulin signaling pathways in the hippocampus. Furthermore, CSF/blood glucose, inflammatory response or acetyl cholinesterase enzyme (AChE) activity were reduced by berberine. Additionally, acetylcholine levels were enhanced after berberine treatment in diabetic rats. Finally, Aβ formation in diabetic hippocampus was inhibited and spatial learning memory was ameliorated by berberine.

**Discussion:** In conclusion, berberine clears Aβ deposit and consequently ameliorates spatial learning memory impairment *via* the activation of the cholinergic anti-inflammatory and insulin signaling pathways in diabetic rats.

## Introduction

Diabetes mellitus (DM) is a chronic and widespread metabolic disease characterized as a consequence of both genetic predisposition and dietary indiscretion and has high prevalence rates and mortality worldwide (Bhusal et al., 2014). Accumulating evidence suggests that Alzheimer’s disease (AD)-like alteration in DM is a major public health problem (Carvalho et al., 2012). Spatial learning and memory impairment induced by streptozotocin in high-fat diet-induced DM animal models is a consequence of changes in the central nervous system (CNS). Diabetes-induced β-amyloid (Aβ) accumulation promotes cognitive impairment involving several pathogenesis (Ohno et al., 2004; Moreira, 2013; Jayaraman and Pike, 2014). From a neuropathological point of view, the senile plaques caused by Aβ, synapse loss, and apoptosis in the neurons are the principal pathological hallmarks of AD. However, the upstream molecular mechanism of diabetes that leads to Aβ deposit is still worth exploring.

Chronic inflammation infiltration plays an important role in cognitive impairment caused by diabetes (Stranahan et al., 2016). Impaired cholinergic system also leads to cognitive impairment as recently reported by various research groups (Ferreira-Vieira et al., 2016). The alpha-7 nicotinic cholinergic receptor subunit (α7nAChRs), which is derived from nicotinic cholinergic receptor, possesses the potential of anti-inflammation in the periphery and CNS. This action is specifically called “the cholinergic anti-inflammatory pathway (CAP)” (Pavlov and Tracey, 2006; Gallowitsch-Puerta and Pavlov, 2007). α7nAChRs are widely distributed throughout CNS and in multiple inflammatory cells, including mononuclear macrophages (Yi et al., 2015), T lymphocytes (Liu et al., 2014), B lymphocytes (Koval et al., 2018) and natural killer (NK) cells (Zanetti et al., 2016). Additionally, α7nAChRs are also abundantly expressed in astrocytes, which are the key regulators of neuroinflammation in several neurodegenerative diseases (Di Cesare Mannelli et al., 2015; Revathikumar et al., 2016). Activated α7nAChRs blocks NF-κB signaling pathway and reduces neuroinflammation (Patel et al., 2017). In the median prefrontal cortex of diabetic rats, activated astrocytes and inflammatory mediators are remarkably increased (Saravia et al., 2002). Thus, astrocytes were considered as emerging pivotal regulators to CNS inflammatory responses in our model (Sofroniew, 2015). Insulin resistance is caused by the inflammatory response, and inflammation is modulated by the a7nAChR as reported in many experiments (Xu et al., 2012; Li et al., 2016). Moreover, the inflammatory and insulin signaling pathways always intertwine with each other. The mistaken process of Aβ precursor protein (APP) due to insulin resistance has been mentioned in many literatures (Hiltunen et al., 2012). It is possible that inflammatory factor may modify the course of APP to accelerate Aβ deposit *via* insulin resistance promotion.

Berberine, an isoquinoline alkaloid, is purified from *Coptis chinensis* Franch and has attracted notable attention for its powerful capabilities as antioxidant, hypoglycemic, cholesterol-lowering, and acetyl cholinesterase enzyme (AChE) inhibitory effect in the periphery (Hsu et al., 2013; Yu et al., 2018). However, the mechanisms of alleviating spatial learning and memory impairment in CNS, especially in the hippocampus, are still poorly understood. Our previous studies confirmed that berberine (187.75 mg/kg/day) can ameliorate emotional memory decline, but the spatial learning and memory impairment which is induced by diabetes still need more efforts to uncover the veils.

Here, we hypothesized that a7nAChR loss in hippocampus is involved in “CAP” deficit, and excessive inflammatory response may lead to insulin signaling inactivation and cognitive impairment in DM rats. This study aimed to investigate the molecular mechanism of berberine in relieving inflammatory effects and modulating the cholinergic and insulin signaling pathways to improve spatial learning and memory impairment in DM rats.

## Materials and Methods

### Reagents

Monoclone antibodies IR, PI3K P85, p-NF-κB p65, IKK, BACE-1, APP, α7nAChR and polyclone antibody Aβ were purchased from Abcam (Cambridge, MA, USA). Monoclone antibody p-Akt (Ser473), AKT, NF-κB, p-IKK, p-IRS-1(Ser307), and IRS-1 were purchased from Cell Signaling Technology (Boston, MA, USA). Insulin ELISA kit (EZRMI-13) and PVDF membrane (0.45 µm) were obtained from Millipore (Billerica, MA, USA). The cytokines of IL-1β, IL-18 and TNF-α were purchased from BOSTER (Wuhan, China) and the ACh kits (A105-1: tissue, A105-2: Serum) and the AChE kits (A024) were purchased from Nanjing Jiancheng Bioengineering Institute (Nanjing, China). The ladder marker was obtained from Thermo Scientific (Waltham, MA, USA). Finally, the GLU kit was purchased from Shanghai Mind Bioengineering Co., Ltd. (Shanghai, China). Berberine was obtained from Shanghai Yuanye Bio-Technology Co., Ltd. (99% pure, Shanghai, China). All other reagents purchased from located market were of analytical grade.

### Animal and Experimental Procedure

**Figure 1:**
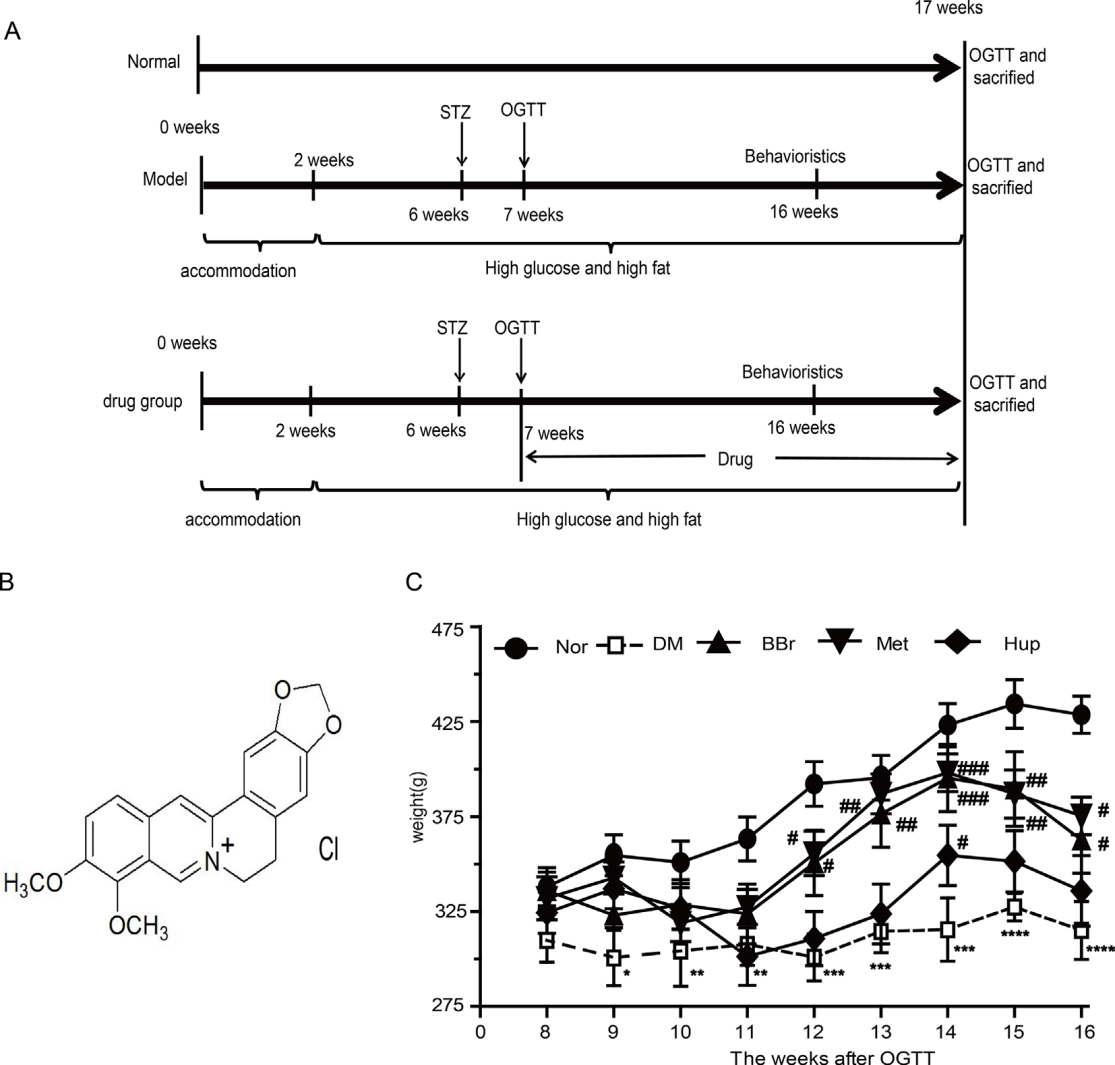
Flow chart of animal experiment design and body weight variation. **(A)** A total of 100 male Wistar rats fed with HSFD diet were assigned to DM model groups, whereas 20 rats fed by standard chow were assigned to control group. After 2 weeks adaptive feeding, DM model group was given HSFD feeding for 4 weeks. Then, 25 mg/kg STZ was injected into caudal vein to form the diabetes animal model in HSFD group, whereas normal group were injected with citrate buffer only. Behavioral evaluation was performed after feeding for 16 weeks. In the 16th week, all rats were sacrificed from which we collected samples and executed follow-up experiment. **(B)** Molecular formula of berberine. **(C)** Body weight variation. *P < 0.05 vs. Nor; **P < 0.01 vs. Nor; ***P < 0.001 vs. Nor; ****P < 0.0001 vs. Nor; ^#^ *P* < 0.05 vs. DM; ^##^ *P* < 0.01 vs. DM; ^###^ *P* < 0.01 vs. DM.

Male Wistar rats weighting 180–200 g (aged 4–5 weeks) were obtained from Vital River Laboratory Animal Technology co., LTD. (Beijing, China). Animals were raised SPF circumstances with a light/dark cycle of 12/12 h under controlled temperature room. Animal experimental protocols were guided and approved in accordance with all guidelines and regulations of the Animal Care and Use Committee affiliated to Tongji Medical College, Huazhong University of Science and Technology. After 2 weeks of adaptation, except for the normal control group (Nor), high sugar and fat diet (HSFD, 67.5% standard laboratory rat chow, 20% sugar, 10% lard, 2% cholesterol and 0.5% bile salts) was given to the DM group for 4 weeks. Following the previous method in our laboratory (Chen et al., 2017), 25 mg/kg Streptozocin (STZ) was injected through the caudal vein in the HSFD group to form the diabetes animal model. After a week feeding, oral glucose tolerance test (OGTT) was used to appraise whether the model was successfully established. Meanwhile, the rats with a blood glucose levels reaching 11.2 after the meal for 2 h were randomly divided into four experimental groups as follows: DM group (DM), berberine group (BBr, 187.75 mg/kg/day), metformin group (Met, 184 mg/kg/day) and huperzine-A group (Hup, 0.015 mg/kg/day). All drugs were prepared avoiding degradation under conditions used due to long time preservation. The rats in Nor group were given standard rodent diet and water *ad libitum*, and those in the STZ-treated group were fed with HSFD until the time for sacrifice. Behavioral test was conducted before the sacrificed week. At the end of the 17th week, body weight was tracked (**Figure 1C**), and glucose level was detected using the commercial glucometer. A timeline of experimental procedure is presented in **Figure 1A**.

### Behavioral Tests

#### Morris Water Maze (MWM)

To evaluate the cognitive impairment and the therapeutic effect of berberine in DM rats, we adopted the Morris water maze (MWM) assay, which was described 20 years ago as a device to investigate spatial learning and memory, and has become one of the most frequently used laboratory tools in behavioral neuroscience(D’hooge and De Deyn, 2001). Testing was conducted *via* the platform-relocation protocol (Izco et al., 2014) with minor modification. The experimental apparatus consists of a circular pool (diameter: 160 cm, depth: 60 cm) filled with water (22–25°C). The water was added with black ink to make it opaque. A black circular platform with 11 cm diameter was placed either 1–2 cm above or submerged 1–2 cm below the water surface. Animals were trained in two consecutive daily cue sessions (platform visible) followed by four acquisition sessions (platform submerged). The pool was located in a small obscured room illuminated by a dim light. All the trials were automatically monitored with a camera above 2 m from water level. MWM assay and data analysis were conducted by unwitting observer.

### Sample Preparation

#### Serial Collection of Cerebrospinal Fluid and Serum

Rat was anesthetized through the intraperitoneal injection of phenobarbital sodium and kept at 37 °C during anesthesia induction. Blood was subsequently collected from abdominal aorta, centrifuged at 3,500 rpm for 10 min to obtain serum, and then stored at −80 °C until for further analysis. Next, the rat’s head placed on the stereotaxic instrument was handled at the neck and dorsum into roughly 130°, and neck skin was shaved. A sagittal incision of the skin was immediately made as inferior to the occiput, whereas foramen magnum was exposed after subcutaneous tissues and muscles were separated through blunt dissection with forceps. A 100 µl syringe was penetrated into the foramen magnum through the dura mater approximately 2–4 mm, and 100 µl of CSF was collected after carefully drawing the piston handle.

#### Hippocampus Sample Preparation

Brains were quickly removed after collecting CSF and rinsed in ice-cold saline. Each set of 6 brains was embedding in paraffin after being fixed by 4% paraformaldehyde. After being dissected on a cold plate, a small portion of the hippocampus tissue was used for enzyme activity detection. The remaining hippocampus was used for Western-blot, carefully dissected, flash frozen in liquid nitrogen, and preserved at −80 °C until use.

### Biochemical Measurements

#### Glucose and Insulin Levels

The insulin and glucose levels in serum and CSF were analyzed by the rat’s insulin sandwich ELISA kit and glucose detection kit according to the manufacturer’s protocol.

#### TNF-α, IL-1, and IL-18 Measurements

Over-expression of diversified inflammation interleukin has been considered in DM. The levels of TNF-α, IL-1, and IL-18 were measured using ELISA kit as per manufacturer’s instructions.

#### ACh Level and Acetyl Cholinesterase Activity

The cholinergic nerve fibers originating from the forebrain project to the hippocampus is essential to learning and memory. The cholinergic system dysfunction was assessed by measuring the Ach level and AChE activity. According to the commercial biochemical kit, the ACh level and AChE activities in the centrifugal serum and homogenized supernatants coming from hippocampus tissues were measured using a spectrophotometer through a micro plate reader (Synergy 2, USA) as per manufacturer’s protocol.

### Western Blot

Hippocampus lysate approximately 30–40 µg samples were denatured and loaded onto sodium dodecyl sulfate-polyacrylamide gels. Proteins were electrophoretically transferred to a PVDF membrane (Millipore, 0.45 µm). After blocking for 1 h with 5% BSA, the bands were incubated overnight at 4°C with primary antibodies and then with Odyssey fluorescence secondary antibodies for 1 h, washed, and visualized by scanning *via* the Gene apparatus.

### Positron-Emission Tomography (PET) Imaging

PET imaging was performed *via* Trans-PET^®^ BioCaliburn^®^ 700 (Raycan Technology Co., Ltd, Suzhou, China). Rats were fasted overnight and injected with 200 μl 18.5 MBq 18F-FDG (18F-fluorodeoxyglucose) *via* the tail vein. After 60-min uptake periods, the rats were scanned 20 min by a Trans-PET scanner under 2% isoflurane anesthesia. Then, PET images were reconstructed using the 3D OSEM algorithm (1 iteration and 12 subsets). We quantified the 18F-FDG uptake in different brain regions of interest (BROI) through the semi-quantitative analysis. The following equation: $\mathrm{SUV}=C_{T}\cdot \frac{V_{T}}{W_{T}}\;\cdot \frac{1}{D_{\mathrm{Inj}}}\cdot \;W_{S}$ was adopted to calculate the standard uptake value (SUV) for each BROI. CT is the radioactivity with the unit of mCi/cc tissue. VT and WT express the volume and weight, respectively. Their ratio generates the density of the region. DInj is the dose injected with the unit of mCi. WS is the weight of the rat in the unit of g.

### IHC Staining and Congo Red Staining

The slices were immunochemically stained as per previous studies (Wang et al., 2018). Images were obtained using a fluorescence microscope (Olympus).

Congo red staining with minor modification (Labour et al., 2016) was adopted to appraise Aβ deposit. Images were obtained with a fluorescence microscope (Olympus).

### Statistical Analysis

Data were collected and analyzed using Graph Pad Prism 5 software and expressed as mean ± SEM. Statistical significance was tested *via one-way* ANOVA following *Tukey’s Post-Test*. *P* values < 0.05 indicated that the levels of significance existed.

## Results

### Berberine Improves the Glucose Absorption and Metabolism in DM Rats

**Figure 2:**
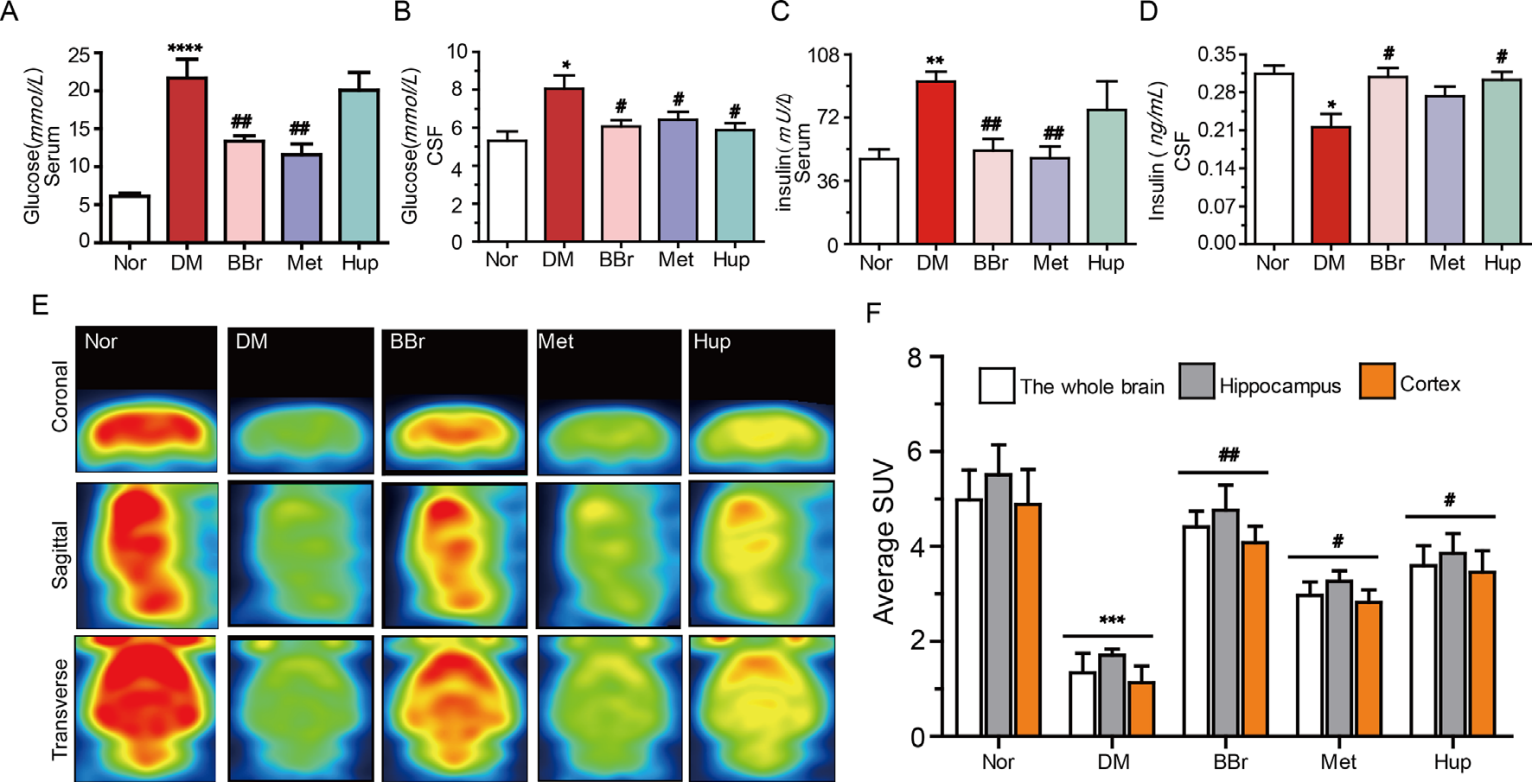
Berberine improves glucose absorption and metabolism in diabetic rats. **(A**, **B)** Glucose levels in diabetic rat’s periphery and CSF, n = 8–12. **(C**, **D)** The insulin levels in diabetic rat’s periphery and CSF, n = 8–12. **(E**, **F)** The glucose metabolism in the brain were measured by PET imaging and quantified by fluorescence intensity, n = 3. *P < 0.05 vs. Nor; **P < 0.01 vs. Nor; ***P < 0.001 vs. Nor; ****P < 0.0001 vs. Nor; ^#^ *P* < 0.05 vs. DM; ^##^ *P* < 0.01 vs. DM

Glucose intolerance and impairment of insulin secretion are associated with a high risk to develop dementia or AD (Ronnemaa et al., 2008). Insulin resistance in DM is typically characterized by peripheral hyperinsulinemia. Dysfunction of glucose metabolism in the CNS caused by peripheral hyperinsulinemia has always been a mystery. On this basis, we measured the glucose and insulin levels in the peripheral and CSF, respectively. We also probed the potential mechanisms of berberine (the structure is as shown in **Figure 1B**) to improve glucose transport. As shown in **Figure 2 A**, the postprandial blood glucose in peripheral was substantially increased after STZ injection combined with HSFD diet. Berberine or metformin remarkably reduced the glucose level, whereas huperzine-A (an AChE inhibitor) did not have an effect. Although the glucose level was lower in CSF than in serum, the variation tendency of glucose levels was consistent with serum results (**Figure 2B**). Furthermore, the serum insulin level was significantly increased in DM rats but was normalized *via* berberine treatment (**Figure 2C**). However, the insulin level in CSF was remarkably decreased in DM rats, and berberine or huperzine-A can increase its level (**Figure 2D**). Interestingly, metformin reduced insulin level in the serum rather than the CSF in DM rats (**Figures 2C, D**). Next, we used PET imaging to detect the brain’s ability to metabolize glucose. As shown in **Figures 2E, F**, glucose transport ability in hippocampus and cortex were significantly impeded in DM rats. This phenomenon was partly reversed by the administration of berberine and huperzine-A. Metformin slightly increased the transport of glucose. In summary, insulin resistance occurs in the diabetic brain, and berberine may facilitate glucose absorption and utilization in DM rats *via* the amelioration of insulin resistance.

### Berberine Alleviates Inflammation in DM Rats

**Figure 3:**
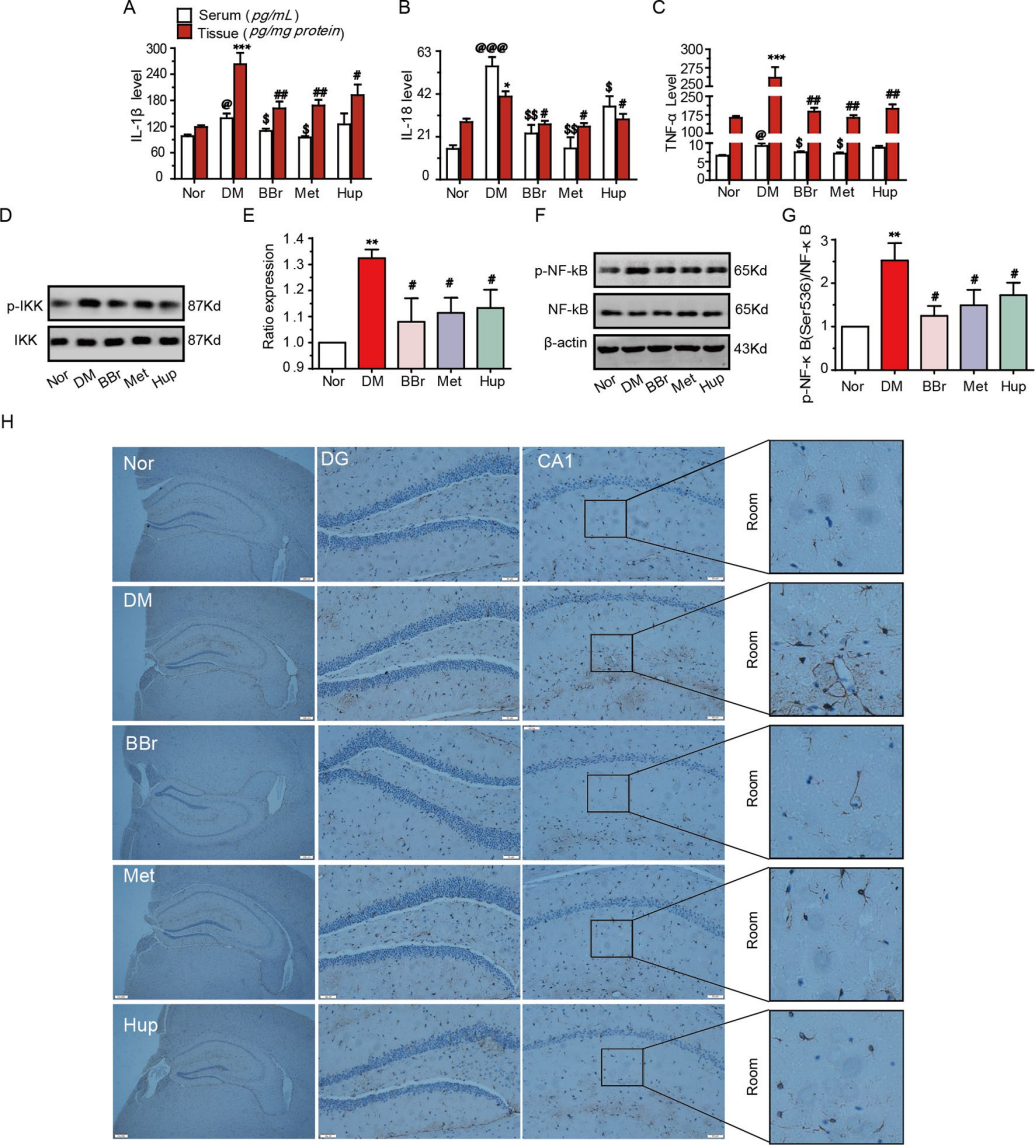
Berberine inhibited inflammatory response in diabetic rats. The IL-1β **(A)**, IL-18 **(B)** and TNF-a **(C)** levels were measured using ELISA kits, n = 8–12. Inflammatory pathway related molecular p-IKK/IKK **(D**, **E)** and p-NF-kB/NF-Kb **(F**, **G)** levels were detected by Western-blot, n = 3. The GFAP expression was appraised by IHC **(H)**, n = 3. *P < 0.05 vs. Nor; **P < 0.01 vs. Nor; ***P < 0.001 vs. Nor; ^@^ *P* < 0.05 vs. Nor; ^@@@^ *P* < 0.001vs. Nor; ^#^ *P* < 0.05 vs. DM; ^##^ *P* < 0.01 vs. DM; ^$^ *P* < 0.05 vs. DM; ^$$^ *P* < 0.01 vs. DM.

The dysfunction of glucose transport and metabolism in the peripheral or CNS results in systemic or local inflammation, which contributes to DM development (Jorge et al., 2011; Muriach et al., 2014). The inflammatory response in the median prefrontal cortex of diabetic rats impairs the fear memory (Chen et al., 2017). Our results demonstrated that the level of IL-1β, IL-18, and TNF-α were significantly increased in the serum and hippocampus tissue in DM rats, whereas this trend was reversed by treatment with berberine, metformin, and huperzine-A (**Figures 3A–C**). To investigate anti-inflammatory effects of berberine, we examined the phosphorylation levels of NF-κB and IKK in hippocampal tissues. As shown in **Figures 3D–G**, p-IKK and p-NF-κB were significantly increased in the hippocampus in DM rats, suggesting that NF-κB pathway may be activated. This phenomenon can be partly inhibited by treatment with berberine, metformin, and huperzine-A. We used a GFAP marker to identify astrocyte activation in hippocampal tissues and further elucidate the origin of inflammation. The activated astrocytes in DM rats were remarkably attenuated by berberine, metformin, and huperzine-A (**Figure 3H**). Thus, berberine may ameliorate hippocampus inflammation *via* the inhibition of astrocytes activation in DM rats.

### Berberine Ameliorates “CAP” Dysfunction in DM Rats

**Figure 4:**
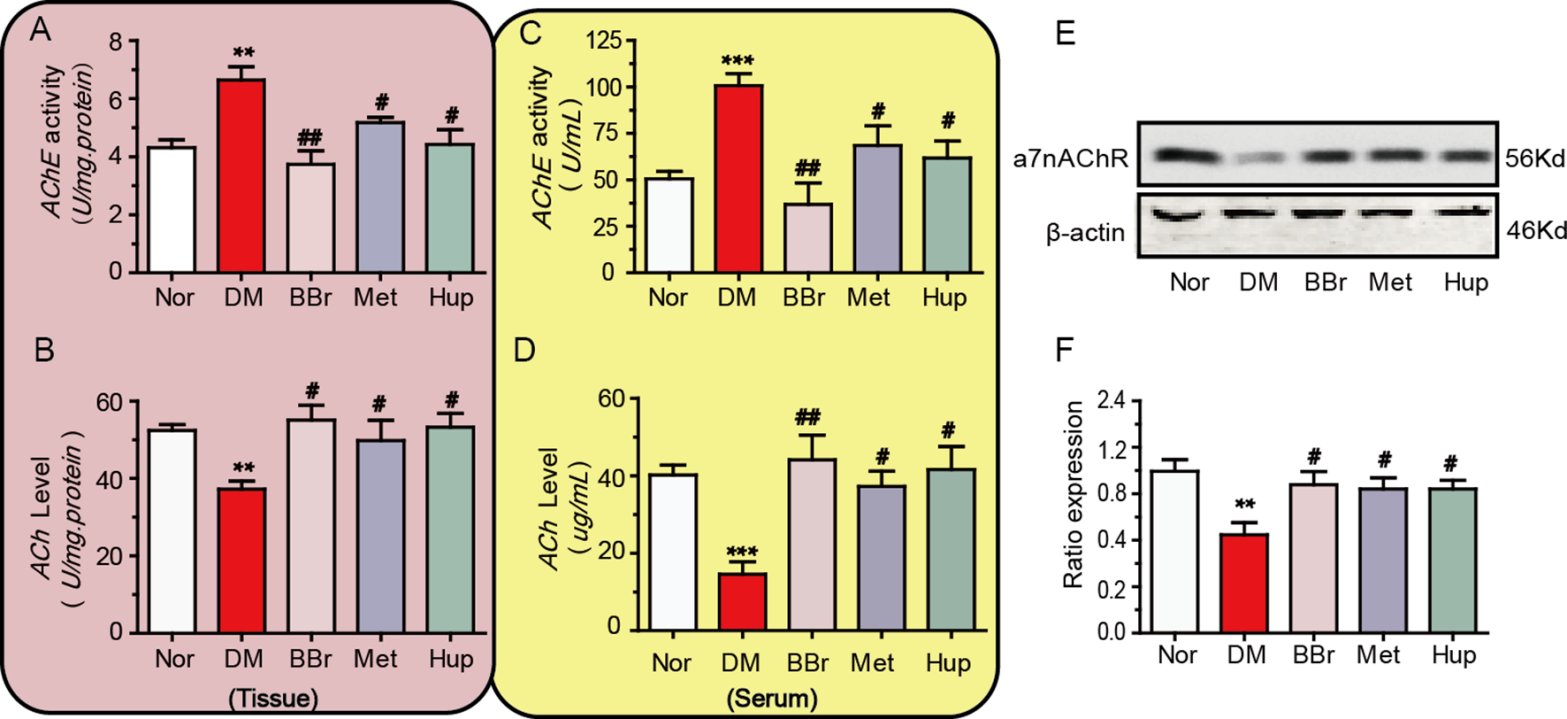
Berberine ameliorates the cholinergic signaling pathway dysfunction. **(A**, **B)** The AChE activity and ACh levels in diabetic rat’s periphery, n = 8–12. **(C**, **D)** The AChE activity and ACh levels in diabetic rat’s hippocampus, n = 8–12. **(E**, **F)** The a7nAChR expression in diabetic rat’s hippocampus was detected by Western-blot and quantified by image J, n = 3. **P < 0.01 vs. Nor; ***P < 0.001 vs. Nor; ^#^ *P* < 0.05 vs. DM; ^##^ *P* < 0.01 vs. DM.

“CAP” plays an important role in the development of systemic and local inflammation. Therefore, we detected the key molecules of the cholinergic signaling pathway within peripheral serum and hippocampus of DM rats. The AChE activity in hippocampal tissues and serum were significantly increased in DM rats, and treatment with berberine, metformin, and huperzine-A decreased the AChE activity (**Figures 4A, C**). The ACh levels in DM rats were increased by the treatment with berberine, metformin, and huperzine-A (**Figures 4B, D**). We further detected a7nAchR expression to assess the anti-inflammatory role of berberine *via* “CAP.” Results demonstrated that a7nAchR expression was remarkably down regulated in the hippocampus of DM rats, but was restored *via* the treatment with berberine, metformin, or huperzine-A (**Figures 4E, F**).

### Berberine Improves the Disorder of Insulin Signaling Pathway in Diabetic Rat’s Hippocampus

**Figure 5:**
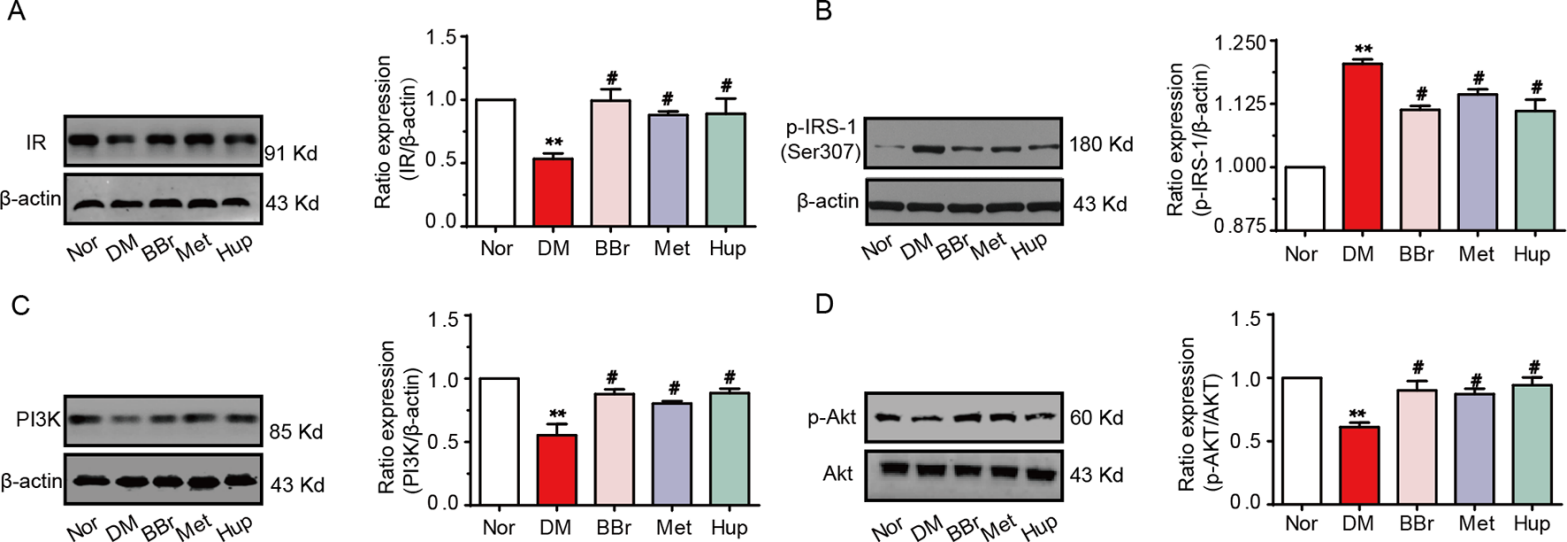
Berberine improves insulin signaling pathway. Western-blot was performed to evaluate the protein expression of IR **(A)**, p-IRS-1 ser307 **(B)**, PI3K **(C)** and p-Akt **(D)** in hippocampus, n = 3. **P < 0.01 vs. Nor; ^#^ *P* < 0.05 vs. DM.

Disorders of the inflammatory signaling pathway trigger insulin resistance. We found that the insulin level in the CSF was decreased and the glucose level was increased, suggesting that the insulin signaling pathway may be dysregulated in the hippocampus of DM rats. Thus, we analyzed the changes of insulin signaling pathways in hippocampus. The insulin receptor (IR) expression was significantly down regulated in DM rats, but was recovered by berberine, metformin, and huperzine-A treatment (**Figure 5A**). In addition, the expression of p-IRS-1 at ser307 in DM rats was notably upregulated, whereas it was partly decreased after treatment with berberine, metformin, and huperzine-A (**Figure 5B**). Next, we detected the expression of PI3K/AKT (key downstream proteins in the insulin signaling pathway). Results revealed that PI3K and p-Akt expression were significantly down regulated in DM rats, but were normalized by berberine, metformin, and huperzine-A (**Figures 5C, D**), suggesting that berberine may improve insulin resistance in the hippocampus of DM rats.

### Berberine Improves APP Misprocessing in the Hippocampus of Diabetic Rats

**Figure 6:**
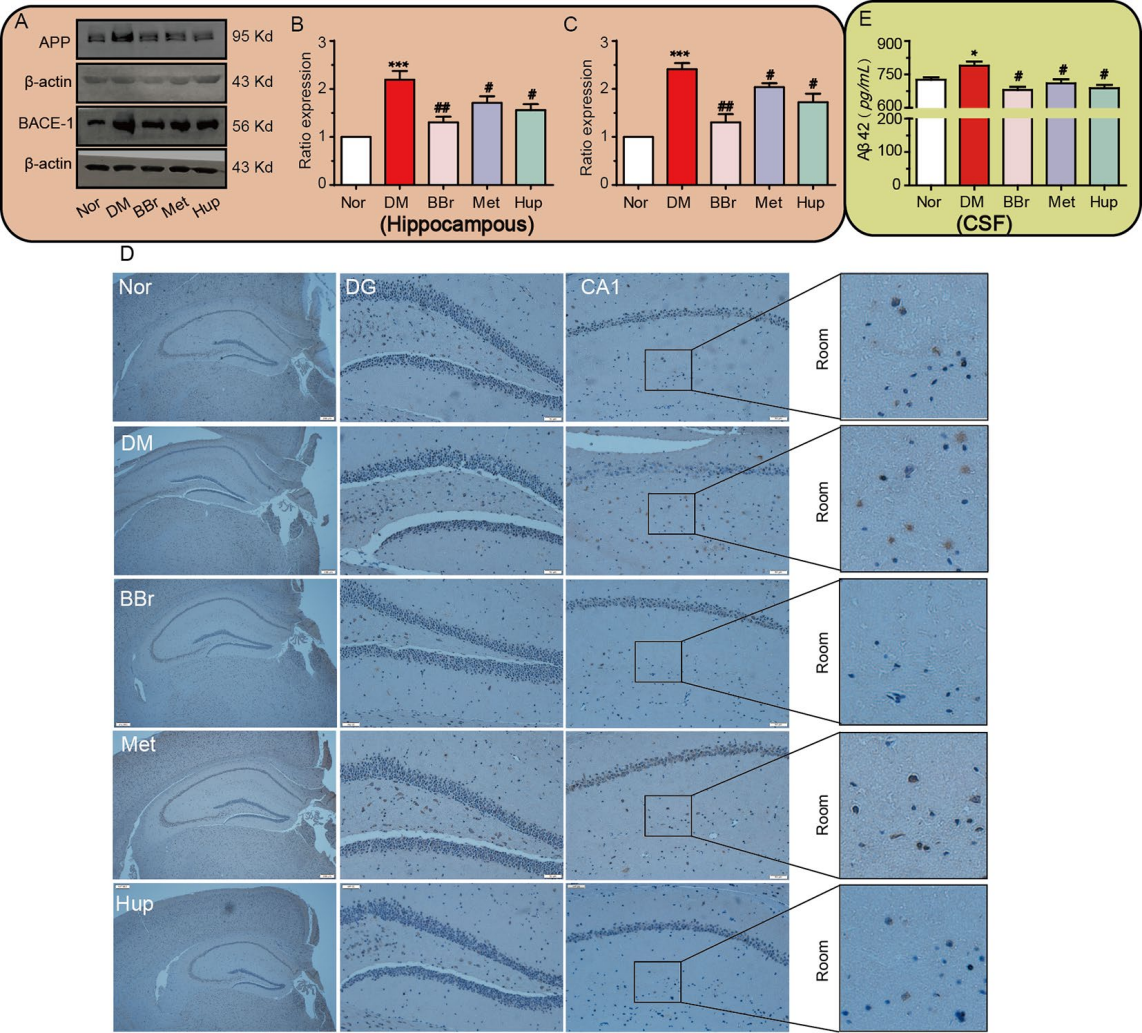
Berberine improves APP misprocessing in the hippocampus. **(A**–**C)** APP and BACE-1 expressions were assessed by Western-blot, n = 3. The senile plaque formation is evaluated by Congo red staining, n = 3 **(D)**. Aβ 42 levels in CSF were detected using ELISA kits, n = 8–12**(E)**. *P < 0.05 vs. Nor; ***P < 0.001 vs. Nor; ^#^ *P* < 0.05 vs. DM; ^##^ *P* < 0.01 vs. DM.

Aβ is always tangled with insulin signaling pathway in diabetic encephalopathy and stagnates insulin receptor in the cytosol. We confirmed that the increased APP/BACE-1 in the hippocampus of DM rats was significantly inhibited by the three compounds (**Figures 6A–C**). Next, to further detect Aβ deposits in DM hippocampal tissue, Congo red staining was applied to assess Aβ generation. Obviously, dark red particles in the DM group increased, but were significantly improved by berberine, metformin, and huperzine A treatment (**Figure 6D**). The level of Aβ42 in CSF was also significantly increased in DM rats, whereas was strongly reduced after drug treatment (**Figure 6E**).

### Berberine Ameliorates the DM-Induced Cognitive Impairment

**Figure 7:**
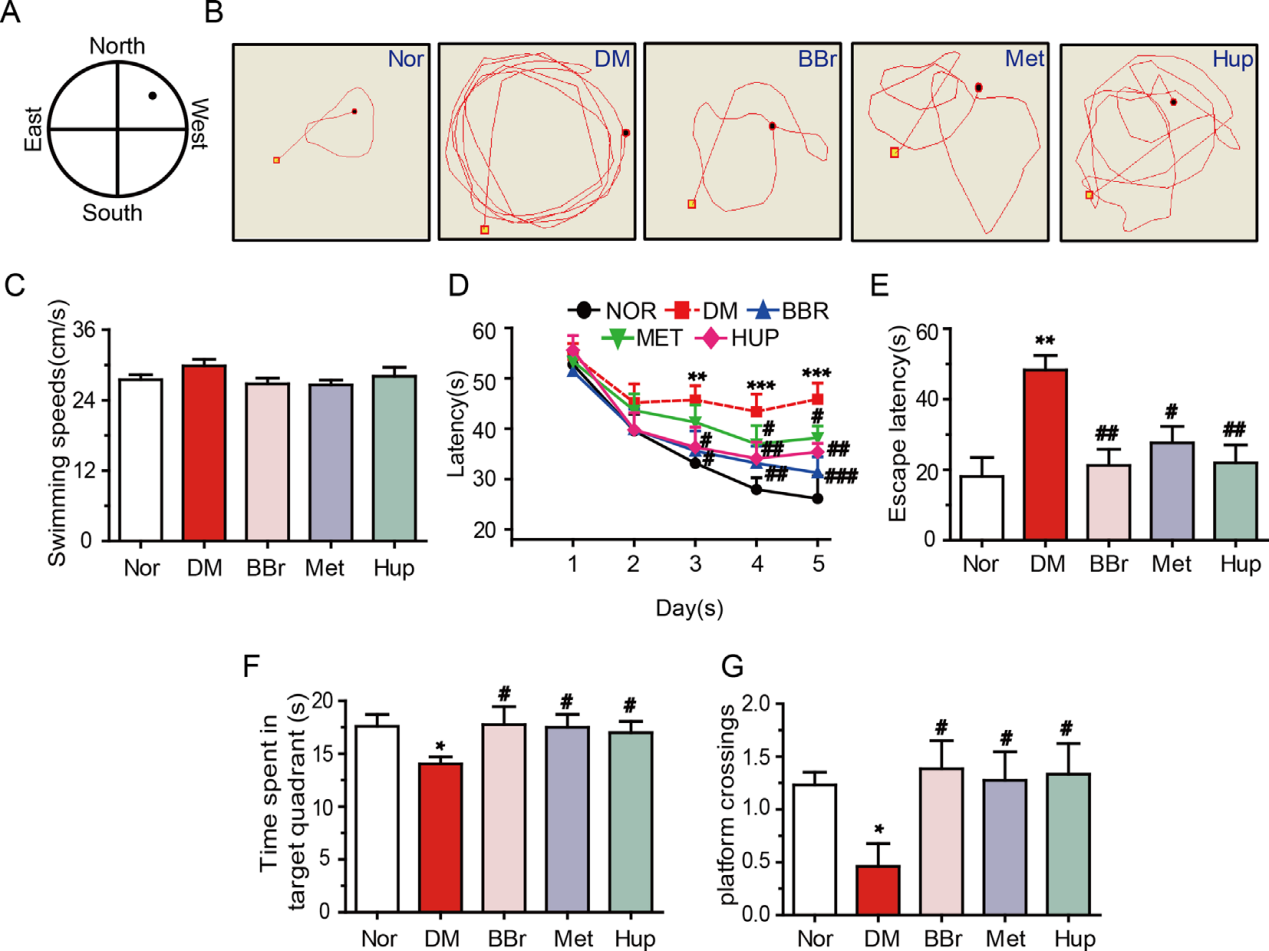
Berberine ameliorates DM-induced cognitive impairment. **(A)** MWM schematic. **(B)** The track of rats in MWM. The swimming speed **(C)** and escape latency analysis in the navigation training trials **(D**, **E)**. Time spent in target quadrant **(F)** and platform crossing **(G)** in the training trials. n = 8–12. *P < 0.05 vs. Nor; **P < 0.01 vs. Nor; ***P < 0.001 vs. Nor; ^#^ *P* < 0.05 vs. DM; ^##^ *P* < 0.01 vs. DM; ^###^ *P* < 0.01 vs. DM.

Cognitive impairment is positively correlation with DM (Luchsinger, 2012). Thus, we analyzed the changes of learning and memory in DM rats using MWM test. Results demonstrated that the escape latency that traveled to the target was significantly retarded in DM rats (**Figures 7A, B**). Spatial learning in DM rats was significantly damaged, although the average swimming speed in 6 days was not changed (**Figures 7C, D**). Besides, escape latencies within 6 days were remarkably increased compared with those in the control group, suggesting that DM rats exhibited deficiency in memory recall (**Figure 7E**). Fortunately, berberine, metformin, and huperzine-A treatment groups had significantly decreased time to reach the platform compared with the DM rats (**Figure 7E**). Additionally, berberine, metformin, and huperzine-A significantly increased the time spent in target quadrant and the number of platform crossings when compared with the DM rats (**Figures 7F, G**). These results suggest that berberine may improve spatial learning and memory impairment in DM rats, so did by metformin and huperzine-A.

## Discussion

DM is rapidly growing among the population and patients have higher incidences of cognitive impairment (Ryan et al., 2014; Infante-Garcia et al., 2018). DM-associated cognitive deficit complication is also referred to as DE (diabetic encephalopathy). Meanwhile, AD and DM share several similar molecular processes, which suggests that both may have common pathological characteristics (Mushtaq et al., 2015). Insulin dysfunction, hyperglycemia, impaired cholinergic system, and inflammation in DM may affect synaptic plasticity, learning and memory, and ultimately result in AD. Berberine has versatile functions, such as hypoglycemic, cholesterol-lowing, anti-bacterial, anti-inflammation, clearance of oxygen radical, and a well-documented effect against memory deficit (Kumar et al., 2015; Cicero and Baggioni, 2016). However, the molecular mechanism and the effectiveness of treatment require more exploration studies. Our data showed that berberine mainly ameliorated spatial learning and memory dysfunction *via* alleviating cholinergic neurological disorders in this study.

It has been acknowledged that DM shows hyperglycemia/hyper-insulin in peripheral tissues (Joubert et al., 2018). Thus, we detected the fasting blood glucose/insulin level and found that berberine significantly decreased the elevated peripheral blood glucose/insulin in DM rats. Simultaneously, our results demonstrated that insulin levels in CSF were reduced in DM rats, whereas were normalized by the hypoglycemic agents such as berberine or metformin and the cholinesterase inhibitor huperzine-A. Furthermore, we measured the level of 18F-FDG *in vivo* and found that the transport of glucose from the periphery to the CNS maybe significantly reduction, and which also indicated that the cell activities were inadequate in the brain. The reason for this may be that DM-induced cerebral vascular disease impairs the blood brain barrier (BBB) and glucose application impairment. Many documents have reported chronic hyperglycemia, which resulted in thickening of the capillary basement membrane in the BBB and narrowing of the lumen (Williamson et al., 1988; Carlson et al., 2003). Moreover, lipid metabolism disturbance is a common occurrence in diabetic patients, which results in higher blood viscosity and lower blood flow (Cho et al., 2008). Hence, these may impair saturation transport mechanism of glucose and insulin, and reduce cerebral blood flow. However, long-term effects will inevitably affect the glucose metabolism of neuron (Marioni et al., 2010; Noh et al., 2014; Antunes et al., 2015). Neurons are highly dependent on glucose energy supply. The impairment on glucose metabolism of neuron accelerates disease deterioration and DE formation and studies should adopt this factor to illustrate the detailed mechanism. Fortunately, berberine can attenuate the loss of glucose transport or activate the cell activity, and this effect is similar to those of huperzine-A and metformin. These results demonstrated that berberine may facilitate glucose metabolism to maintain normal brain function.

Neuroinflammation contributes to the initiation and subsequent development of neurodegenerative disorders. In the brain, two types of glial cells, astroglia and microglia, are major contributors to neuroinflammatory processes (Tribouillard-Tanvier et al., 2012). Thus, microglia has always been considered as professional macrophage or resident immune cell within the brain. Astroglia, which represents most abundant glial cell population, has recently attracted considerable attention for studies on crucial neuroinflammatory processes. Astroglia activation produces numerous cytokines, such as IL-1β, and IL-6 (Wilson et al., 2002; Sofroniew, 2015; Dhanda et al., 2018). Increasing evidence suggests that berberine can inhibit inflammatory response in peripheral tissue of diabetes (Jeong et al., 2009; Zhou et al., 2009; Amasheh et al., 2010), but no reports on the anti-inflammatory activities of berberine in CNS have been documented. Our results exhibit that IL-1β, IL-18 and TNF-α levels in serum and hippocampus were notably increased in DM rats. The phosphorylation of IKK and NF-KB, the downstream molecules of inflammatory signaling pathways, were also upregulated in hippocampus. We also observed that GFAP was significantly increased in the ventral region of the hippocampus, suggesting that astroglia was activated in the hippocampus of DM rats. Berberine reduced the GFAP expression, cytokine levels, IKK and NF-KB phosphorylation, implying that this drug may attenuate inflammation by inhibiting astroglia activation in the hippocampus of DM rats. The “CAP” regulates inflammation mainly through the ACh-α7nAChR interaction. Interestingly, CNS or peripheral inflammation can be inhibited by the activation of a7nAChR (Revathikumar et al., 2016; Lu et al., 2017; Kong et al., 2018). In the peripheral blood and hippocampus, berberine increased ACh level and inhibited AChE activity in DM rats. Meanwhile, we found that berberine also upregulated α7nAChRs expression in the hippocampus of DM rats. These results suggested that berberine may improve the cholinergic signaling pathway, which is equivalent to the cholinesterase inhibitor huperzine A.

Numerous epidemiological and experimental studies showed that individuals with diabetes have a higher risk of developing AD, providing a substantial link between DM and AD (Jimenez-Palomares et al., 2012). Compelling evidence supported that accumulation of Aβ42 can accelerate cognitive impairment in double-transgenic mice (Aso et al., 2015; Guo et al., 2015). Given that AChE induced Aβ fibril formation (Pradhan et al., 2018), as in previous experiments, we have confirmed that berberine reduces the erroneous processing of APP in mPFC. On this basis, we can infer that berberine fulfills its protective effects by lessening Aβ deposition. To clarify the mechanism, we examined the related molecules of Aβ-produced in diabetic hippocampus. Remarkable up-regulation of APP, BACE-1 and Aβ42 were observed in the hippocampus. After treatment with berberine, metformin or huperzine-A, these elevated trends were effectively suppressed. Similarly, Congo red staining was used to further verify that Aβ42 deposit response in the ventral hippocampus of diabetic rats was enhanced. The changes of Aβ42 deposit were attenuated after the drug treatment. Collectively, our findings demonstrated that berberine has the potential of removing Aβ42.

Long-term insulin resistance in brain leads to the formation of Aβ plaque and development of AD, and this phenomenon also appear in DM (Kim and Feldman, 2015). Conversely, impaired cholinergic system (Okamoto and Nishimura, 2015), formation of tau protein hyperphosphorylation (Wang et al., 2018) and pro-inflammatory events (Petersen and Shulman, 2018) were all conducive to insulin signaling deficit. Insulin resistance and massive Aβ appeared in the medial prefrontal cortex of diabetic rats in our previous data, which were ameliorated by berberine (Chen et al., 2017). In our results, reference learning and procedural memory were impairment in DM rats. The escape latency in positioning navigation experiments was significantly prolonged, and the time spent in the target exploration area in the space exploration experiment was significantly shortened. This finding fully explained the impairment of the maintenance of spatial learning and memory in DM rats. Fortunately, berberine effectively improves cognitive impairment. Collectively, current data showed that berberine alleviates the inflammatory, cholinergic and insulin signaling deficits in diabetic hippocampal tissue, and exhibits a protective effect on spatial learning and memory impairment.

It is well known that metformin was used to cure T2DM through the amelioration of insulin resistance. Huperzine-A, an inhibitor of AChE, is usually used to treat AD. Several studies and the present results showed that metformin can reverse diabetes-induced cognitive impairment. In our previous studies, metformin showed anti-inflammation effect, but this function is not through “GAP,” which indicates that another signaling pathway participates in the anti-inflammation function. Huperzine-A can activate “GAP” by inhibiting AChE and increasing a7nAChR expression in the CNS. However, Huperzine-A cannot inhibit inflammation generation and have no attenuation to insulin resistance in the periphery. A number of compounds in Chinese herbal medicine exhibit attractive potency involving the modulation of intracellular signaling pathways of inflammation, such as flavonoids (Chen et al., 2018), alkaloids (Chang, 2017), polyphenols (Teng and Chen, 2018), and terpenoids (Teng et al., 2018). It is well known that berberine shows its safety and efficiency in several clinical trials and can be used to treat various metabolic diseases, such as cardiovascular diseases (Feng et al., 2019), nonalcoholic hepatic steatosis (Zhu et al., 2019), nervous systems diseases (Jiang et al., 2015), and kidney disease (Qin et al., 2019). In our studies, berberine can not only activate “GAP” in the CNS, but also activate in the periphery. Besides, berberine alleviates insulin resistance, and modulates glucose metabolism in the CNS and periphery.

**Figure 8:**
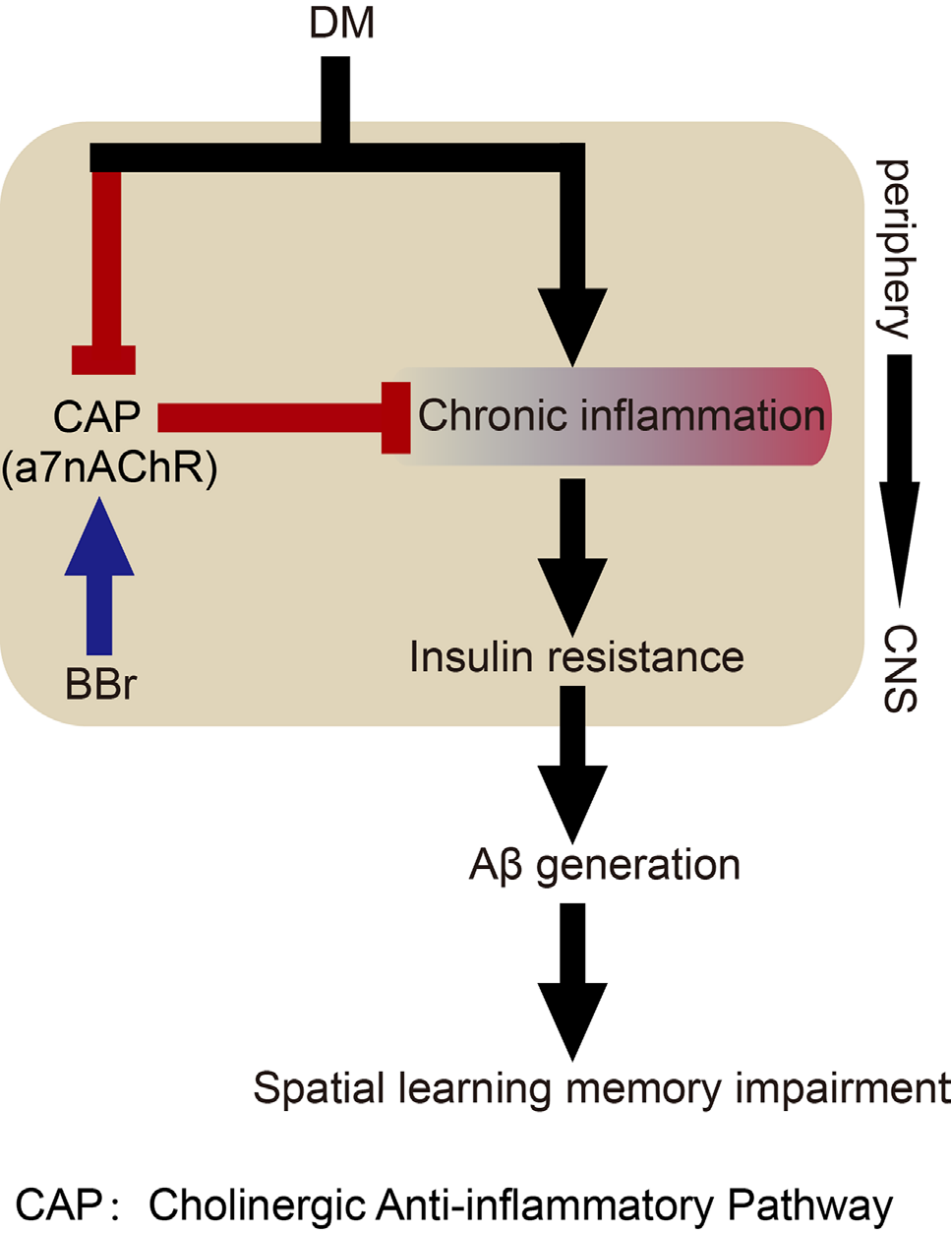
Hypothetical mechanism of berberine to ameliorate impairment of spatial learning and memory in DM rats.

In conclusion, our study uncovers that berberine may primarily inhibit astrocyte activation by affecting glucose metabolism and cholinergic disturbance in the periphery and CNS, thereby alleviating inflammatory response and insulin resistance, and finally ameliorate spatial learning and memory impairment (**Figure 8**). The versatility of berberine may help us to elucidate the mechanism in diabetes-induced cognitive impairment through crosstalk between signaling pathways.

## Data Availability

All datasets generated for this study are included in the manuscript.

## Ethics Statement

The study protocol was in agreement with Animal Care and Use Committee affiliated to the Huazhong University of Science and Technology.

## Author Contributions

KW and QC performed most of the experiments. NW and YL participated in data analyses and the discussion of the manuscript. RZ, JW, XZ, and DG collected the samples. CL and JC participated in its design and coordination. All authors read and approved the final manuscript.

## Funding

This work was supported by the National Natural Science Foundation of China (81573785, 81373872) to KW, the National Natural Science Foundation of China (81371416, 31670778, 81873725), and the Fundamental Research Funds for the Central Universities (HUST: 2016YXMS191, 2014YGYL005) to JC.

## Conflict of Interest Statement

The authors declare that the research was conducted in the absence of any commercial or financial relationships that could be construed as a potential conflict of interest.

## Abbreviations

DM, diabetes mellitus; AD, Alzheimer’s disease; Aβ, β-amyloid; APP, amyloid precursor protein; BACE-1, beta-site amyloid precursor protein cleaving enzyme 1; STZ, streptozocin; HSFD, high sugar and fat diet; OGTT, oral glucose tolerance test; 18F-FDG, 18F-fluorodeoxyglucose; PET, Positron-Emission Tomography; IHC, Immunohistochemical.

## References

[B1] Amasheh M. Fromm A. Krug S. M. Amasheh S. Andres S. Zeitz M. (2010). TNFalpha-induced and berberine-antagonized tight junction barrier impairment *via* tyrosine kinase, Akt and NFkappaB signaling. J. Cell. Sci. 123, 4145–4155. 10.1242/jcs.070896 21062898

[B2] Antunes H. K. De Mello M. T. Santos-Galduroz R. F. Galduroz J. C. Lemos V. A. Tufik S. (2015). Effects of a physical fitness program on memory and blood viscosity in sedentary elderly men. Braz. J. Med. Biol. Res. 48, 805–812. 10.1590/1414-431x20154529 26222648 PMC4568808

[B3] Aso E. Sanchez-Pla A. Vegas-Lozano E. Maldonado R. Ferrer I. (2015). Cannabis-based medicine reduces multiple pathological processes in AbetaPP/PS1 mice. J. Alzheimers Dis. 43, 977–991. 10.3233/JAD-141014 25125475

[B4] Bhusal A. Jamarkattel N. Shrestha A. Lamsal N. K. Shakya S. Rajbhandari S. (2014). Evaluation of antioxidative and antidiabetic activity of bark of holarrhena pubescens wall. J. Clin. Diagn. Res. 8, Hc05–Hc08. 10.7860/JCDR/2014/7803.4863 PMC4225906 25386454

[B5] Carlson E. C. Audette J. L. Veitenheimer N. J. Risan J. A. Laturnus D. I. Epstein P. N. (2003). Ultrastructural morphometry of capillary basement membrane thickness in normal and transgenic diabetic mice. Anat. Rec. A Discov. Mol. Cell. Evol. Biol. 271, 332–341. 10.1002/ar.a.10038 12629676

[B6] Carvalho C. Cardoso S. Correia S. C. Santos R. X. Santos M. S. Baldeiras I. (2012). Metabolic alterations induced by sucrose intake and Alzheimer’s disease promote similar brain mitochondrial abnormalities. Diabetes 61, 1234–1242. 10.2337/db11-1186 22427376 PMC3331754

[B7] Chang W. (2017). Non-coding RNAs and Berberine: a new mechanism of its anti-diabetic activities. Eur. J. Pharmacol. 795, 8–12. 10.1016/j.ejphar.2016.11.055 27915042

[B8] Chen L. Teng H. Jia Z. Battino M., (2018). Intracellular signaling pathways of inflammation modulated by dietary flavonoids: the most recent evidence. Crit. Rev. Food Sci. Nutr. 58, 2908–2924. 10.1080/10408398.2017.1345853 28682647

[B9] Chen Q. Mo R. Wu N. Zou X. Shi C. Gong J. (2017). Berberine Ameliorates Diabetes-Associated Cognitive Decline through Modulation of Aberrant Inflammation Response and Insulin Signaling Pathway in DM Rats. Front. Pharmacol. 8, 334. 10.3389/fphar.2017.00334 28634451 PMC5460072

[B10] Cho Y. I. Mooney M. P. Cho D. J. (2008). Hemorheological disorders in diabetes mellitus. J. Diabetes Sci. Technol. 2, 1130–1138. 10.1177/193229680800200622 19885302 PMC2769810

[B11] Cicero A. F. Baggioni A. (2016). Berberine and Its Role in Chronic Disease. Adv. Exp. Med. Biol. 928, 27–45. 10.1007/978-3-319-41334-1_2 27671811

[B12] D’hooge R. De Deyn P. P. (2001). Applications of the Morris water maze in the study of learning and memory. Brain Res. Brain Res. Rev. 36, 60–90. 10.1016/S0165-0173(01)00067-4 11516773

[B13] Dhanda S. Gupta S. Halder A. Sunkaria A. Sandhir R. (2018). Systemic inflammation without gliosis mediates cognitive deficits through impaired BDNF expression in bile duct ligation model of hepatic encephalopathy. Brain Behav. Immun. 70, 214–232. 10.1016/j.bbi.2018.03.002 29518527

[B14] Di Cesare Mannelli L. Tenci B. Zanardelli M. Failli P. Ghelardini C. (2015). alpha7 Nicotinic Receptor Promotes the Neuroprotective Functions of Astrocytes against Oxaliplatin Neurotoxicity. Neural Plast. 2015, 396908. 10.1155/2015/396908 26146570 PMC4469839

[B15] Feng X. Sureda A. Jafari S. Memariani Z. Tewari D. Annunziata G. (2019). Berberine in Cardiovascular and Metabolic Diseases: from Mechanisms to Therapeutics. Theranostics 9, 1923–1951. 10.7150/thno.30787 31037148 PMC6485276

[B16] Ferreira-Vieira T. H. Guimaraes I. M. Silva F. R. Ribeiro F. M. (2016). Alzheimer’s disease: targeting the Cholinergic System. Curr. Neuropharmacol. 14, 101–115. 10.2174/1570159X13666150716165726 26813123 PMC4787279

[B17] Gallowitsch-Puerta M. Pavlov V. A. (2007). Neuro-immune interactions *via the* cholinergic anti-inflammatory pathway. Life Sci. 80, 2325–2329. 10.1016/j.lfs.2007.01.002 17289087 PMC2921074

[B18] Guo H. B. Cheng Y. F. Wu J. G. Wang C. M. Wang H. T. Zhang C. (2015). Donepezil improves learning and memory deficits in APP/PS1 mice by inhibition of microglial activation. Neuroscience 290, 530–542. 10.1016/j.neuroscience.2015.01.058 25662507

[B19] Hiltunen M. Khandelwal V. K. Yaluri N. Tiilikainen T. Tusa M. Koivisto H. (2012). Contribution of genetic and dietary insulin resistance to Alzheimer phenotype in APP/PS1 transgenic mice. J. Cell. Mol. Med. 16, 1206–1222. 10.1111/j.1582-4934.2011.01384.x 21762376 PMC3823075

[B20] Hsu Y.-Y. Tseng Y.-T. Lo Y.-C. (2013). Berberine, a natural antidiabetes drug, attenuates glucose neurotoxicity and promotes Nrf2-related neurite outgrowth. Toxicol. Appl. Pharmacol. 272, 787–796. 10.1016/j.taap.2013.08.008 23954465

[B21] Infante-Garcia C. Ramos-Rodriguez J. J. Hierro-Bujalance C. Ortegon E. Pickett E. Jackson R., (2018). Antidiabetic Polypill Improves Central Pathology and Cognitive Impairment in a Mixed Model of Alzheimer’s Disease and Type 2 Diabetes. Mol. Neurobiol. 55, 6130–6144. 10.1007/s12035-017-0825-7 29224179

[B22] Izco M. Martinez P. Corrales A. Fandos N. Garcia S. Insua D. (2014). Changes in the brain and plasma Abeta peptide levels with age and its relationship with cognitive impairment in the APPswe/PS1dE9 mouse model of Alzheimer’s disease. Neuroscience 263, 269–279. 10.1016/j.neuroscience.2014.01.003 24447596

[B23] Jayaraman A. Pike C. J. (2014). Alzheimer’s disease and type 2 diabetes: multiple mechanisms contribute to interactions. Curr. Diab. Rep. 14, 476. 10.1007/s11892-014-0476-2 24526623 PMC3985543

[B24] Jeong H. W. Hsu K. C. Lee J. W. Ham M. Huh J. Y. Shin H. J. (2009). Berberine suppresses proinflammatory responses through AMPK activation in macrophages. Am. J. Physiol. Endocrinol. Metab. 296, E955–E964. 10.1152/ajpendo.90599.2008 19208854

[B25] Jiang W. Li S. Li X. (2015). Therapeutic potential of berberine against neurodegenerative diseases. Sci. China Life Sci. 58, 564–569. 10.1007/s11427-015-4829-0 25749423 PMC5823536

[B26] Jimenez-Palomares M. Ramos-Rodriguez J. J. Lopez-Acosta J. F. Pacheco-Herrero M. Lechuga-Sancho A. M. Perdomo G. (2012). Increased Abeta production prompts the onset of glucose intolerance and insulin resistance. Am. J. Physiol. Endocrinol. Metab. 302, E1373–E1380. 10.1152/ajpendo.00500.2011 22414803

[B27] Jorge M. L. De Oliveira V. N. Resende N. M. Paraiso L. F. Calixto A. Diniz A. L. (2011). The effects of aerobic, resistance, and combined exercise on metabolic control, inflammatory markers, adipocytokines, and muscle insulin signaling in patients with type 2 diabetes mellitus. Metabolism 60, 1244–1252. 10.1016/j.metabol.2011.01.006 21377179

[B28] Joubert M. Manrique A. Cariou B. Prieur X. (2018). Diabetes-related cardiomyopathy: the sweet story of glucose overload from epidemiology to cellular pathways. Diabetes Metab. 45. 10.1016/j.diabet.2018.07.003 30078623

[B29] Kim B. Feldman E. L. (2015). Insulin resistance as a key link for the increased risk of cognitive impairment in the metabolic syndrome. Exp. Mol. Med. 47, e149. 10.1038/emm.2015.3 25766618 PMC4351418

[B30] Kong W. Kang K. Gao Y. Liu H. Meng X. Cao Y. (2018). GTS-21 Protected Against LPS-Induced Sepsis Myocardial Injury in Mice Through alpha7nAChR. Inflammation 41, 1073–1083. 10.1007/s10753-018-0759-x 29680908

[B31] Koval L. Kalashnyk O. Lykhmus O. Skok M. (2018). alpha7 nicotinic acetylcholine receptors are involved in suppression of the antibody immune response. J. Neuroimmunol. 318, 8–14. 10.1016/j.jneuroim.2018.01.012 29395323

[B32] Kumar A. Ekavali Chopra K. Mukherjee M. Pottabathini R. Dhull D. K. (2015). Current knowledge and pharmacological profile of berberine: an update. Eur. J. Pharmacol. 761, 288–297. 10.1016/j.ejphar.2015.05.068 26092760

[B33] Labour M. N. Vigier S. Lerner D. Marcilhac A. Belamie E. (2016). 3D compartmented model to study the neurite-related toxicity of Abeta aggregates included in collagen gels of adaptable porosity. Acta Biomater 37, 38–49. 10.1016/j.actbio.2016.04.001 27057929

[B34] Li F. Zhao Y. B. Wang D. K. Zou X. Fang K. Wang K. F. (2016). Berberine relieves insulin resistance *via the* cholinergic anti-inflammatory pathway in HepG2 cells. J. Huazhong Univ. Sci. Technol. Med. Sci. 36, 64–69. 10.1007/s11596-016-1543-5 26838742

[B35] Liu Z. Han B. Li P. Wang Z. Fan Q. (2014). Activation of alpha7nAChR by nicotine reduced the Th17 response in CD4(+)T lymphocytes. Immunol. Invest. 43, 667–674. 10.3109/08820139.2014.914532 24949556

[B36] Lu X. X. Hong Z. Q. Tan Z. Sui M. H. Zhuang Z. Q. Liu H. H. (2017). Nicotinic Acetylcholine Receptor Alpha7 Subunit Mediates Vagus Nerve Stimulation-Induced Neuroprotection in Acute Permanent Cerebral Ischemia by a7nAchR/JAK2 Pathway. Med. Sci. Monit. 23, 6072–6081. 10.12659/MSM.907628 29274273 PMC5747934

[B37] Luchsinger J. A. (2012). Type 2 diabetes and cognitive impairment: linking mechanisms. J. Alzheimers Dis. 30 Suppl 2, S185–S198. 10.3233/JAD-2012-111433 22433668 PMC3372666

[B38] Marioni R. E. Deary I. J. Strachan M. W. Lowe G. D. Rumley A. Murray G. D. (2010). Blood rheology and cognition in the Edinburgh Type 2 Diabetes Study. Age Ageing 39, 354–359. 10.1093/ageing/afq021 20197283

[B39] Moreira P. I. (2013). High-sugar diets, type 2 diabetes and Alzheimer’s disease. Curr. Opin. Clin. Nutr. Metab. Care 16, 440–445. 10.1097/MCO.0b013e328361c7d1 23657152

[B40] Muriach M. Flores-Bellver M. Romero F. J. Barcia J. M. (2014). Diabetes and the brain: oxidative stress, inflammation, and autophagy. Oxid. Med. Cell. Longev. 2014, 102158. 10.1155/2014/102158 25215171 PMC4158559

[B41] Mushtaq G. Khan J. A. Kumosani T. A. Kamal M. A. (2015). Alzheimer’s disease and type 2 diabetes *via* chronic inflammatory mechanisms. Saudi J. Biol. Sci. 22, 4–13. 10.1016/j.sjbs.2014.05.003 25561876 PMC4281591

[B42] Noh H. J. Seo S. W. Jeong Y. Park J. E. Kim G. H. Noh Y. (2014). Blood viscosity in subcortical vascular mild cognitive impairment with versus without cerebral amyloid burden. J. Stroke Cerebrovasc. Dis. 23, 958–966. 10.1016/j.jstrokecerebrovasdis.2013.08.004 24589034

[B43] Ohno M. Sametsky E. A. Younkin L. H. Oakley H. Younkin S. G. Citron M. (2004). BACE1 deficiency rescues memory deficits and cholinergic dysfunction in a mouse model of Alzheimer’s disease. Neuron 41, 27–33. 10.1016/S0896-6273(03)00810-9 14715132

[B44] Okamoto N. Nishimura T. (2015). Signaling from Glia and Cholinergic Neurons Controls Nutrient-Dependent Production of an Insulin-like Peptide for Drosophila Body Growth. Dev. Cell. 35, 295–310. 10.1016/j.devcel.2015.10.003 26555050

[B45] Patel H. Mcintire J. Ryan S. Dunah A. Loring R. (2017). Anti-inflammatory effects of astroglial alpha7 nicotinic acetylcholine receptors are mediated by inhibition of the NF-kappaB pathway and activation of the Nrf2 pathway. J. Neuroinflammation 14, 192. 10.1186/s12974-017-0967-6 28950908 PMC5615458

[B46] Pavlov V. A. Tracey K. J. (2006). Controlling inflammation: the cholinergic anti-inflammatory pathway. Biochem. Soc. Trans. 34, 1037–1040. 10.1042/BST0341037 17073745

[B47] Petersen M. C. Shulman G. I. (2018). Mechanisms of Insulin Action and Insulin Resistance. Physiol. Rev. 98, 2133–2223. 10.1152/physrev.00063.2017 30067154 PMC6170977

[B48] Pradhan K. Das G. Mondal P., (2018). Genesis of Neuroprotective Peptoid from Abeta30-34 Inhibits Abeta Aggregation and AChE Activity. ACS Chem. Neurosci. 9. 10.1021/acschemneuro.8b00071 30036464

[B49] Qin X. Zhao Y. Gong J. Huang W. Su H. Yuan F. (2019). Berberine Protects Glomerular Podocytes *via* Inhibiting Drp1-Mediated Mitochondrial Fission and Dysfunction. Theranostics 9, 1698–1713. 10.7150/thno.30640 31037132 PMC6485199

[B50] Revathikumar P. Bergqvist F. Gopalakrishnan S. Korotkova M. Jakobsson P. J. Lampa J. (2016). Immunomodulatory effects of nicotine on interleukin 1beta activated human astrocytes and the role of cyclooxygenase 2 in the underlying mechanism. J. Neuroinflammation 13, 256. 10.1186/s12974-016-0725-1 27681882 PMC5041575

[B51] Ronnemaa E. Zethelius B. Sundelof J. Sundstrom J. Degerman-Gunnarsson M. Berne C. (2008). Impaired insulin secretion increases the risk of Alzheimer disease. Neurology 71, 1065–1071. 10.1212/01.wnl.0000310646.32212.3a 18401020

[B52] Ryan J. P. Fine D. F. Rosano C. (2014). Type 2 diabetes and cognitive impairment: contributions from neuroimaging. J. Geriatr. Psychiatry Neurol. 27, 47–55. 10.1177/0891988713516543 24394151 PMC4049175

[B53] Saravia F. E. Revsin Y. Gonzalez Deniselle M. C. Gonzalez S. L. Roig P. Lima A. (2002). Increased astrocyte reactivity in the hippocampus of murine models of type 1 diabetes: the nonobese diabetic (NOD) and streptozotocin-treated mice. Brain Res. 957, 345–353. 10.1016/S0006-8993(02)03675-2 12445977

[B54] Sofroniew M. V. (2015). Astrocyte barriers to neurotoxic inflammation. Nat. Rev. Neurosci. 16, 249–263. 10.1038/nrn3898 25891508 PMC5253239

[B55] Stranahan A. M. Hao S. Dey A. Yu X. Baban B. (2016). Blood–brain barrier breakdown promotes macrophage infiltration and cognitive impairment in leptin receptor-deficient mice. J. Cereb. Blood Flow Metab. 36, 2108–2121. 10.1177/0271678X16642233 27034250 PMC5363667

[B56] Teng H. Chen L. (2018). Polyphenols and Bioavailability: an update. Crit. Rev. Food. Sci. Nutr. 59, 2040–2051. 10.1080/10408398.2018.1437023 29405736

[B57] Teng H. Yuan B. Gothai S. Arulselvan P. Song X. Chen L. (2018). Dietary triterpenes in the treatment of type 2 diabetes: to date. Trends Food Sci. Technol. 72, 34–44. 10.1016/j.tifs.2017.11.012

[B58] Tribouillard-Tanvier D. Carroll J. A. Moore R. A. Striebel J. F. Chesebro B. (2012). Role of cyclophilin A from brains of prion-infected mice in stimulation of cytokine release by microglia and astroglia *in vitro.* J. Biol. Chem. 287, 4628–4639. 10.1074/jbc.M111.269480 22179611 PMC3281647

[B59] Wang S. He B. Hang W. Wu N. Xia L. Wang X. (2018). Berberine Alleviates Tau Hyperphosphorylation and Axonopathy-Associated with Diabetic Encephalopathy *via* Restoring PI3K/Akt/GSK3beta Pathway. J. Alzheimers Dis. 65, 1385–1400. 10.3233/JAD-180497 30175975

[B60] Williamson J. R. Tilton R. G. Chang K. Kilo C. (1988). Basement membrane abnormalities in diabetes mellitus: relationship to clinical microangiopathy. Diabetes Metab. Rev. 4, 339–370. 10.1002/dmr.5610040404 3292174

[B61] Wilson C. J. Finch C. E. Cohen H. J. (2002). Cytokines and cognition—the case for a head-to-toe inflammatory paradigm. J. Am. Geriatr. Soc. 50, 2041–2056. 10.1046/j.1532-5415.2002.50619.x 12473019

[B62] Xu T. Y. Guo L. L. Wang P. Song J. Le Y. Y. Viollet B. (2012). Chronic exposure to nicotine enhances insulin sensitivity through alpha7 nicotinic acetylcholine receptor-STAT3 pathway. PLoS One 7, e51217. 10.1371/journal.pone.0051217 23251458 PMC3520975

[B63] Yi L. Luo J. F. Xie B. B. Liu J. X. Wang J. Y. Liu L. (2015). alpha7 Nicotinic Acetylcholine Receptor is a Novel Mediator of Sinomenine Anti-Inflammation Effect in Macrophages Stimulated by Lipopolysaccharide. Shock 44, 188–195. 10.1097/SHK.0000000000000389 25895149

[B64] YuY.ZhaoY.TengF.LiJ.GuanY.XuJ. (2018). Berberine Improves Cognitive Deficiency and Muscular Dysfunction *via* Activation of the AMPK/SIRT1/PGC-1a Pathway in Skeletal Muscle from Naturally Aging Rats. J. Nutr. Health Aging 22, 710–717. 10.1007/s12603-018-1015-7 29806860

[B65] Zanetti S. R. Ziblat A. Torres N. I. Zwirner N. W. Bouzat C. (2016). Expression and Functional Role of alpha7 Nicotinic Receptor in Human Cytokine-stimulated Natural Killer (NK) Cells. J. Biol. Chem. 291, 16541–16552. 10.1074/jbc.M115.710574 27284006 PMC4974370

[B66] Zhou J. Zhou S. Tang J. Zhang K. Guang L. Huang Y. (2009). Protective effect of berberine on beta cells in streptozotocin- and high-carbohydrate/high-fat diet-induced diabetic rats. Eur. J. Pharmacol. 606, 262–268. 10.1016/j.ejphar.2008.12.056 19374872

[B67] Zhu X. Bian H. Wang L. Sun X. Xu X. Yan H. (2019). Berberine attenuates nonalcoholic hepatic steatosis through the AMPK-SREBP-1c-SCD1 pathway. Free Radic. Biol. Med. 141, 192–204. 10.1016/j.freeradbiomed.2019.06.019 31226399

